# Differences in plasma TIMP-1 levels between healthy people and patients with rectal cancer stage II or III

**DOI:** 10.2478/v10019-011-0027-2

**Published:** 2011-08-26

**Authors:** Irena Oblak, Franc Anderluh, Vaneja Velenik, Barbara Mozina, Janja Ocvirk, Eva Ciric, Natasa Hrovatic Podvrsnik

**Affiliations:** 1 Department of Radiotherapy; 2 Department of Laboratory Diagnostics; 3 Department of Medical Oncology, Institute of Oncology Ljubljana, Ljubljana, Slovenia

**Keywords:** TIMP-1, rectal cancer, healthy blood donors

## Abstract

**Background:**

The purpose of the study was to analyse whether the levels of the tissue inhibitor of matrix metalloproteinases-1 (TIMP-1) are higher in patients with rectal cancer as compared with healthy blood donors.

**Patients and methods.:**

Two hundred and seventeen patients (147 male, 70 female) with histologically confirmed non-metastatic rectal cancer (clinical stage II–III) and 45 healthy blood donors (15 male, 30 female) were included in analysis. Patient’s mean age was 66 years (range: 34–87 years) and healthy blood donor’s mean age was 35 years (range: 18–64 years). Plasma TIMP-1 concentrations were measured with an enzyme-linked immunosorbent assay (ELISA) using commercially available TIMP-1 ELISA kit. Mann-Whitney-test for independent groups was used to assess the differences of plasma TIMP-1 levels and clinicopathological parameters. Two-sided tests were used and the differences at P<0.05 were considered as statistically significant.

**Results:**

Median patients TIMP-1 level was 180 ng/mL (range: 22-538 ng/mL); the mean (±SD) level was 193.7 (79.5) ng/mL. The median healthy blood donors TIMP-1 level was 112 ng/mL (range: 48-211 ng/mL); the mean (±SD) level was 115 (35.7) ng/mL. TIMP-1 levels in patients with rectal cancer were statistically significantly higher than TIMP-1 levels in healthy blood donors (P<0.0001). Significant differences in TIMP-1 levels were not found comparing gender (P=0.43), but in both groups TIMP-1 levels were increased with higher age (P=0.007).

**Conclusions:**

Patients with rectal cancer had statistically significantly higher mean and median TIMP-1 level than healthy blood donors which is in accordance with the results published in other publications. These findings suggest possibility that plasma TIMP-1 levels could be used as new biological markers for early cancer detection.

## Introduction

Tumour aggressiveness and metastatic potential, resulting in a poor prognosis, have been shown to be increased with the activation of matrix metalloproteinases (MMPs) and their tissue inhibitors (TIMPs). They play an important role in the process of degradation of the extracellular matrix and the basal membrane in relation to tumours invasiveness. The activity of the MMPs depends on the balance between the level of the active enzyme and its tissue inhibitor.^1^–^4^ The TIMP family consists of four members: TIMP-1, TIMP-2, TIMP-3 and TIMP-4, which primary act as negative regulators of the degradation process of the extracellular matrix. Latest evidence suggests that they also participate in cell signalling by regulating cell growth, apoptosis, angiogenesis and genomic instability.^3^,^5^,^6^

Many researchers focused on clinical impact of TIMPs in cancer biology by rating tumour tissue TIMP expression or plasma circulating TIMP level. They found out that TIMPs can be useful diagnostic and prognostic markers like other new markers.^3^,^6^–^8^ Most of these studies evaluated TIMP-1 and they recognized it as an important marker for patient management and prognosis in several malignancies such as colorectal, breast, lung, ovarian, bladder carcinoma^3^,^6^ and in several nonmalignant diseases such as asthma, diabetes, cardiovascular and autoimmune diseases.^6^

Elevated plasma TIMP -1 levels were reported to be significantly increased in colon cancer patients compared with healthy population, and they were also associated with advanced disease stage and poor prognosis.^3^,^5^,^6^,^9^–^12^ The aim of this study is to determine whether the TIMP-1 levels are higher in patients with rectal cancer as compared with healthy blood donors.

## Patients and methods

### Patients

Two hundred and seventeen patients (147 male, 70 female) with histologically confirmed non-metastatic rectal cancer (clinical stage II or III) and 45 healthy blood donors (15 male, 30 female) were included in analysis. Patient’s mean age was 66 years (range: 34–87 years) and healthy blood donor’s mean age was 35 years (range: 18–64 years).

### Determination of plasma TIMP-1 level

All blood samples were taken in the same laboratory, and processed and stored with the same procedures. The blood samples were collected in vacutainer EDTA tubes. After centrifugation at 1500×g at 4°C for 10 minutes, plasma was separated and kept at −80°C until analysis. Plasma TIMP-1 concentrations were measured with an enzyme-linked immunosorbent assay (ELISA) using commercially available TIMP-1 ELISA kit, purchased from Oncogene Science, Cambridge (MA), USA. All procedures were performed according to the manufacturer’s protocol in duplicate using diluted samples. Measurements differed by less than 10% and the mean value was calculated and used for statistical analysis. The inter-assay precision coefficients of variation of TIMP-1 ranged between 3.9% and 8.8% at the different levels.

### Statistical analysis

Statistical analysis was performed using personal computer and software statistical package SPSS, version 13 (SPSS Inc., USA). The main endpoint of the study was to determine if rectal cancer patients have higher TIMP-1 levels compared to healthy blood donors. Mann-Whitney-test for independent groups was used to assess the differences of plasma TIMP-1 levels and clinicopathological parameters. Two-sided tests were used and the differences at P<0.05 were considered as statistically significant.

### Ethical consideration

Prior to analysis, all patients and healthy blood donors received detailed oral and written information about the research and procedures, and they signed an informed consent for their plasma analysis for the purposes of the research. The trial was approved by the ethic committees of the Institute of Oncology, Ljubljana, Slovenia and by the National Medical Ethics Committee of Republic of Slovenia and was in agreement with the Declaration of Helsinki.

## Results

### TIMP-1 levels

**FIGURE 1:**
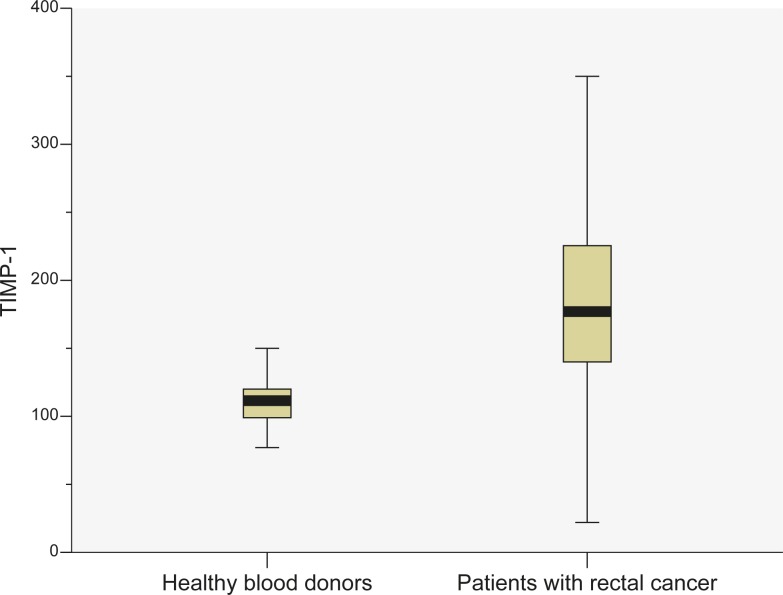
TIMP-1 levels (in ng/mL) in healthy blood donors and in patients.

**FIGURE 2:**
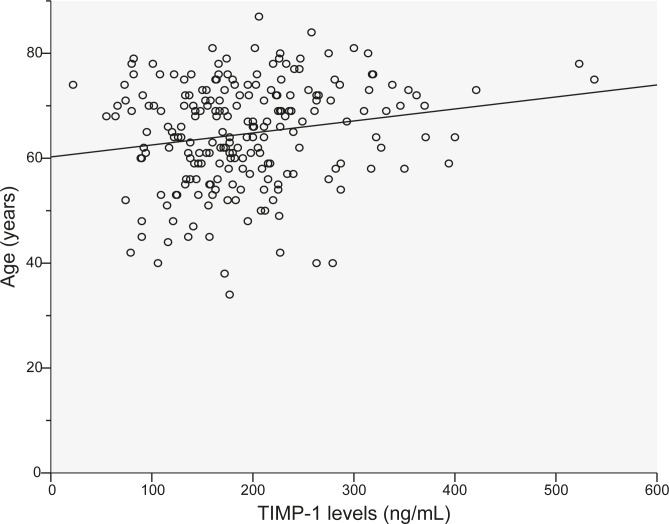
TIMP-1 levels and age in healthy blood donors (p=0.007).

**FIGURE 3:**
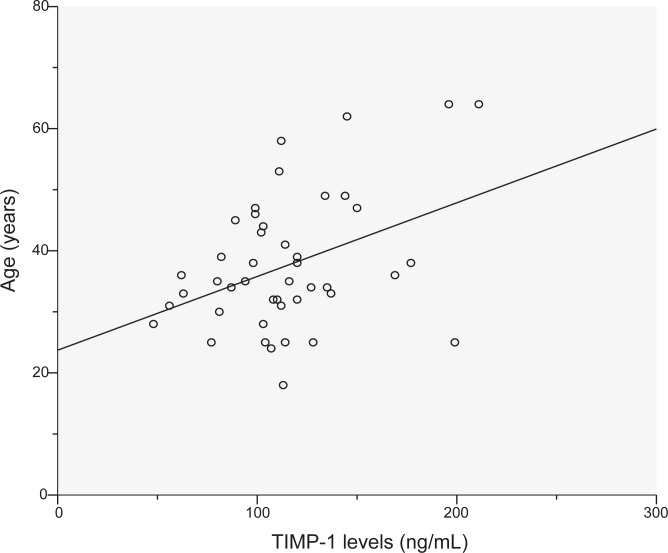
TIMP-1 levels and age in patients (p=0.007).

Median patients TIMP-1 level was 180 ng/mL (range: 22–538 ng/mL); the mean (±SD) level was 193.7 (79.5) ng/mL. The median healthy blood donors TIMP-1 level was 112 ng/mL (range: 48–211 ng/mL); the mean (±SD) level was 115 (35.7) ng/mL (Figure 1). TIMP-1 levels in patients with rectal cancer were statistically significantly higher than TIMP-1 levels in healthy blood donors (P<0.0001). Significant differences in TIMP-1 levels were not found comparing gender (P=0.43), but in both groups TIMP-1 levels were increased with higher age (P=0.007) (Figures 2 and 3).

## Discussion

TIMP-1 plays an important role in regulation of extracellular matrix remodelling, which is one of the processes involved in cancer growth and spread. Several authors reported that patients with malignant disease had significantly higher TIMP-1 levels in comparison with healthy population.^5^,^9^,^11^–^14^ In our study similar results were found. Patients with rectal cancer had statistically significant higher TIMP-1 levels in comparison with healthy blood donors (P<0.0001).

These findings also raise the idea that plasma TIMP-1 measurements could be useful in screening for malignant disease, such as rectal cancer in our case. Future randomised studies will demonstrate if the plasma TIMP-1 could be helpful for screening and detecting colorectal cancer beside digitorectal examination, faecal occult blood testing, recto-, sigmoido- and colonoscopy, DNA stool analysis and genetic testing.^8^,^15^ Screening has in oncology special value as patients with lower stages have better prognosis.^16^

Like other researchers^3^,^12^,^14^ we did not find significant differences in TIMP-1 levels and gender, but Holten-Andersen *et al.*^11^ demonstrated that the mean TIMP-1 levels were approximately 10% higher in males than in females. The reason for this they attributed to the fact that the male population has higher incidence of tromboembolic diseases, which result in higher release rate of TIMP-1 in plasma from activated platelets.^11^

In many studies (in ours as well) TIMP-1 level was found to be increased with age,^3^,^9^,^11^,^14^,^17^ although Tayebjee *et al.* in their study found an inverse relationship between TIMP-1 level and age.^18^

## Conclusions

Patients with rectal cancer had statistically significantly higher mean and median TIMP-1 level than healthy blood donors which is in accordance with the results published in others publications. These findings suggest possibility that plasma TIMP-1 levels could be used as new biological markers for early cancer detection.

## References

[1] Pesta M, Topolcan O, Holubec L, Rupert K, Cerna M, Holubec L (2007). Clinicopathological assesment and quantitative estimation of the matrix metalloproteinases MMP-2 and MMP-7 and the inhibitors TIMP-1 and TIMP-2 in colorectal carcinoma tissue samples. Anticancer Res.

[2] Li M, Yamamoto H, Adachi Y, Maruyama Y, Shinomura Y (2006). Role of matrix metaloproteinase-7 (matrilysin) in human cancer invasion, apoptosis, growth and angiogenesis. Exp Biol Med.

[3] Giaginis C, Nikiteas N, Margeli A, Tzanakis N, Rallis G, Kouraklis G (2009). Serum tissue inhibitor of metalloproteinase 1 and 2 (TIMP-1 and TIMP-2) levels in colorectal cancer patients: association with clinicopathological variables and patients survival. Int J Biol Markers.

[4] Alo-Aho R, Kahari VM (2005). Colagenases in cancer. Biochemie.

[5] Sørensen NM, Sørensen IV, Wűrtz SØ, Schrohl AS, Dowell B, Davis G (2008). Biology and potential clinical implications of tissue inhibitor of metalloproteinases-1 in colorectal cancer treatment. Scan J Gastroenterol.

[6] Frederiksen CB, Lomholt AF, Lottenburger T, Davis GJ, Dowell BL, Blankenstein MA (2008). Assesment of the biological variation of plasma tissue inhibitor of metalloproteinase-1. Int J Biol Markers.

[7] Horvat M, Stabuc B (2011). Microsatellite instability in colorectal cancer. Radiol Oncol.

[8] Yaolin Zhou Y, Boardman LA, Miller RC (2010). Genetic testing for young-onset colorectal cancer: case report and evidence-based clinical guidelines. Radiol Oncol.

[9] Wass ET, Hendriks T, Lomme RM, Woblles T (2005). Plasma levels of matrix metal-loproteinase-2 and tissue inhibitor of metalloproteinase-1 correlate with disease stage and survival in colorectal cancer patients. Dis Colon Rectum.

[10] Kahlert C, Bandapalli OR, Schirmacher P, Weitz J, Brand K (2008). Invasion front-specific overexpression of tissue inhibitor of metalloproteinase-1 in liver metastases from colorectal cancer. Anticancer Res.

[11] Holten-Andersen MN, Murphy G, Nielsen HJ, Pedersen AN, Christensen IJ, Høyer-Hansen G (1999). Quantitation of TIMP-1 in plasma of healthy blood donors and patients with advanced cancer. Br J Cancer.

[12] Hammer JH, Basse L, Svendsen MN, Werther K, Brüner N, Christensen IJ (2006). Impact of elective resection on plasma TIMP-1 in patients with colon cancer. Colorectal Dis.

[13] Holten-Andersen MN, Christensen IJ, Nielsen HJ, Stephens RW, Jensen V, Nielsen OH (2002). Total levels of tissue inhibitor of metalloproteinases 1 in plasma yield high diagnostic sensitivity and specificity in patients with colon cancer. Clin Cancer Res.

[14] Holten-Andersen MN, Stephens RW, Nielsen HJ, Murphy G, Christensen IJ, Stetler-Stevenson W (2000). High preoperative plasma tissue inhibitor of metalloproteinase-1 levels are associated with short survival of patients with colorectal cancer. Clin Cancer Res.

[15] Sofic A, Beslic S, Kocijancic I, Sehovic N (2010). CT colonography in detection of colorectal carcinoma. Radiol Oncol.

[16] Oblak I, Petric P, Anderluh F, Velenik V, Hudej R, Fras AP (2009). Anal cancer chemoirradiation with curative intent - a single institution experience. Neoplasma.

[17] Ishida H, Murata N, Hayashi Y, Tada M, Hashimoto D (2003). Serum levels of tissue inhibitor of metalloproteinases-1 (TIMP- 1) in colorectal cancer patients. Surg Today.

[18] Tayebjee MH, Lip GYH, Blann AD, MacFadyen RJ (2005). Effects of age, gender, ethnicity, diurnal variations and exercise on circulating levels of matrix metalloproteinases (MMP)-2 and -9, and their inhibitors, tissue inhibitors of matrix metalloproteinases (TIMP)-1 and -2. Thromb Res.

